# Supplementary material for: Exploring the cellular landscape of circular RNAs using full-length single-cell RNA sequencing
Source: Nat Commun. 2022 Jun 10;13:3242. doi: 10.1038/s41467-022-30963-8 (PMC9187688; doi:10.1038/s41467-022-30963-8)
Supplement: Supplementary file 1 — Supplementary Information [file 41467_2022_30963_MOESM1_ESM.pdf]

Supplementary Information

Exploring the cellular landscape of circular RNAs using full-length single-cell RNA sequencing

Wanying Wu^, Jinyang Zhang^, Xiaofei Cao, Zhengyi Cai, Fangqing Zhao*

*Corresponding author. Email: zhfq@biols.ac.cn

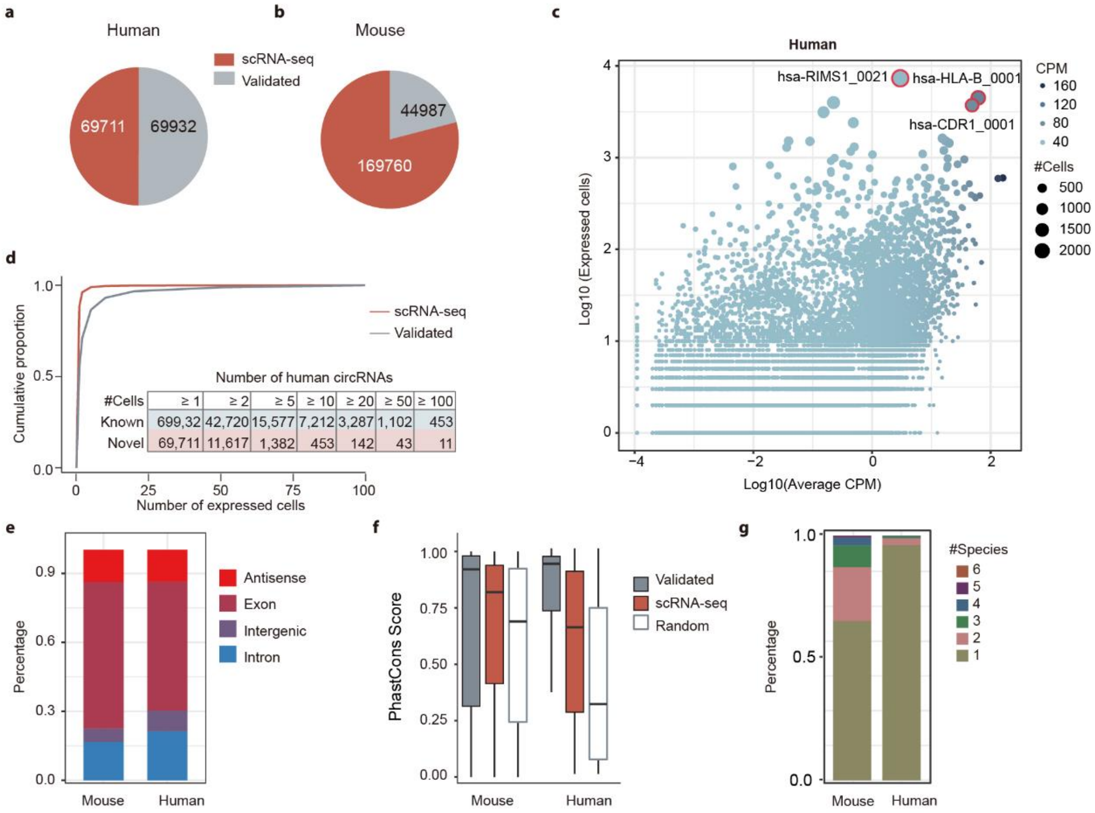

Supplementary Fig. 1 Profiling circRNAs from human and mouse single cell RNA-seq datasets. (a) Overlap of circRNAs detected in human scRNA-seq datasets and public databases. **(b)** Overlap of circRNAs detected in mouse scRNA-seq datasets and public databases. **(c)** Log-scaled mean expression level and the number of expressing cells for human circRNAs only. Sizes of points indicate the number of expressing cells. Filled colors represent the mean CPM of circRNAs. **(d)** Cumulative distribution of human circRNAs ranked by the number of expressing cells. The scRNA-seq specific and known circRNAs are colored in red and grey respectively. **(e)** The proportion of various types of scRNA-seq specific circRNAs in human and mouse cells. **(f)** The average phastCons score of back-spliced exons of scRNA-seq specific circRNAs (n = 85,676 human / 65,242 mouse circRNAs), known circRNAs (n = 12,933 human and 22,679 mouse circRNAs), and randomly selected exons (n = 14,923 human and 17,162 mouse exons). The phastCons data were obtained from the UCSC Genome Browser. **(g)** The number of species that conservatively expressed human and mouse scRNA-seq specific circRNAs. The flanking 100 bp sequence of the back-spliced junction of scRNA-seq specific circRNAs and all circRNAs in bulk RNA-seq based databases were extracted and aligned using BLAT. CircRNAs with ≥ 70 bp matched back-spliced junction sequence were determined as orthologs, and the number of species expressing the orthologs of scRNA-seq specific circRNAs was calculated accordingly. All center lines in the box plots indicate the median values, and box limits indicate the upper and lower quartiles of plotted values. The upper and lower whisker indicate the largest and smallest value within the range of 1.5x IQR from the box limits. Source data are provided as a Source Data file.

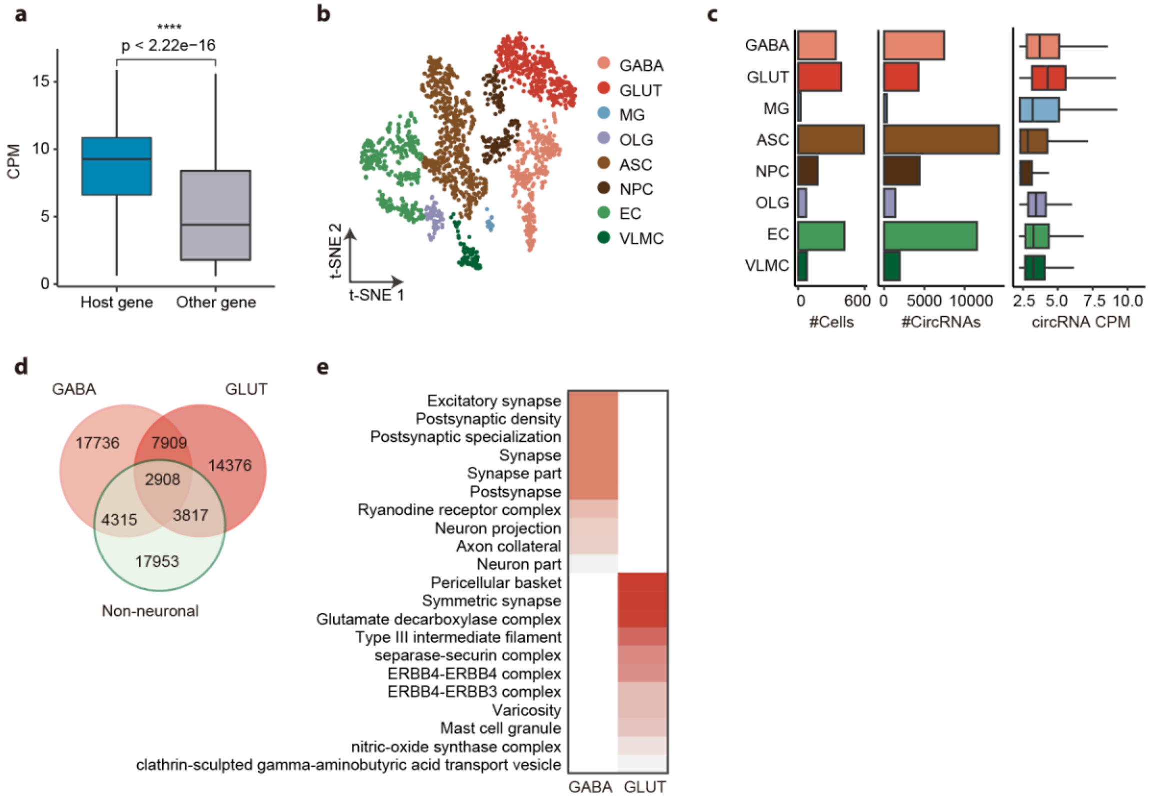

Supplementary Fig. 2 Expression of circRNAs in human and mouse brain cells. (a) The distribution of expression values of the circRNA hosting gene (n = 13,467) and other non-hosting genes (n = 39,253). The y axis represents the normalized expression levels of genes measure by counts per million (CPM). The p value is calculated using two-sided Wilcoxon rank sum test without adjustment for multiple comparisons. **(b)** t-SNE plot based on gene expression profiles of four human brain scRNA-seq datasets, colored by clustered and annotated cell types. **(c)** Number of cells, circRNAs and the expression levels of circRNAs (n = 7,447 / 4,284 / 341 / 14,245 / 4,414 / 1,401 / 11,527 / 1,955 circRNAs) detected in different cell clusters. **(d)** Overlap of circRNAs detected in GABAergic neurons, glutamatergic neurons, and non-neuronal cells. **(e)** Gene ontology enrichment analysis of host genes of GABAergic- and glutamatergic-specific circRNAs. Filled colors indicate the -log10 q-values computed using two-sided Fisher exact test and adjusted using Benjamini-Hochberg method for multiple hypotheses testing. All center lines in the box plots indicate the median values, and box limits indicate the upper and lower quartiles of plotted values. The upper and lower whisker indicate the largest and smallest value within the range of 1.5x IQR from the box limits. **** $P < 0.0001$, Wilcoxon rank-sum test (two-sided). Source data are provided as a Source Data file.

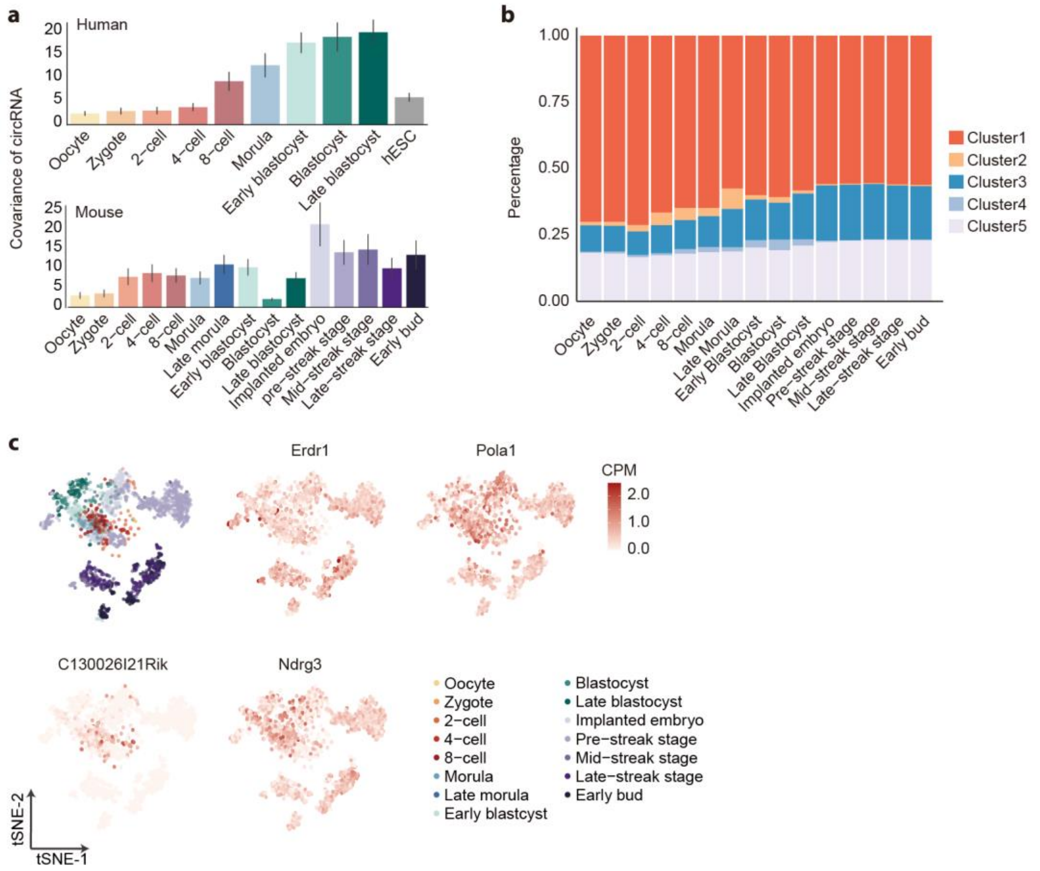

Supplementary Fig. 3 Dynamic changes of circRNAs during embryo development. (a) The covariance of circRNA expression values in the different developing stages of human (upper, n = 5,876 / 11,908 / 13,561 / 18,634 / 16,379 / 22,717 / 4,090 / 9,160 / 2,573 / 2,784 cells) and mouse (bottom, n = 1,928 / 2,579 / 10,998 / 6,789 / 3,174 / 3,262 / 62 / 1,142 / 1,306 / 2,279 / 2,997 / 7,300 / 805 / 1,079 / 1,300 cells) embryos. The upper line of bars indicate the average numbers, and the error bars indicate ± SD of plotted values. **(b)** The number of maternal (cluster 1-2) and zygotic (cluster 3-5) genes in different stages. **(c)** The t-SNE plot of all cells, and expression level of host genes of circRNAs in Fig. 3H. Source data are provided as a Source Data file.

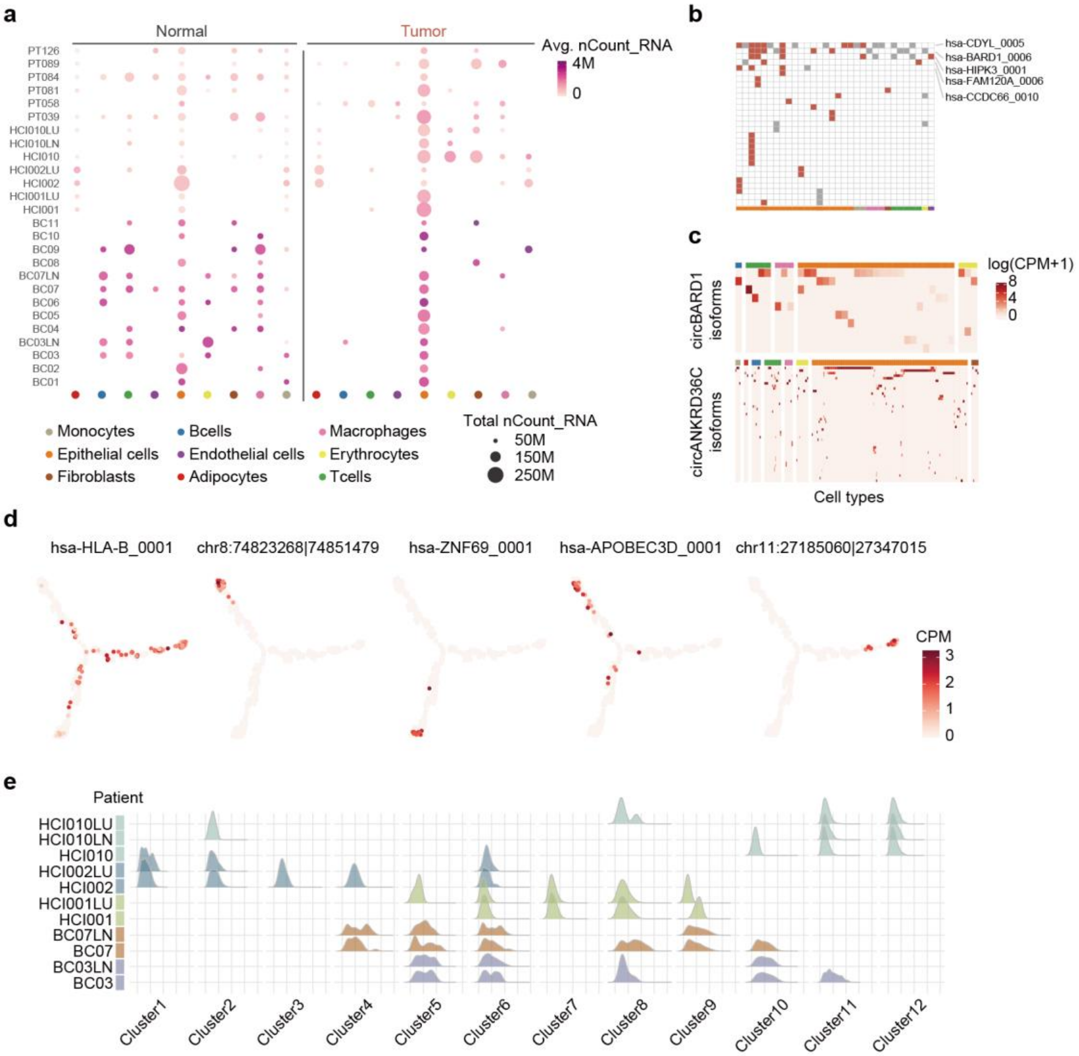

Supplementary Fig. 4 Complexity of circRNAs in normal and tumor epithelial cells. (a) The average library size for different cell types in each patient, measured by *nCount_RNA* from the Seurat results. **(c)** The expression profile of 29 cancer-related circRNAs in different cells. Red and grey color indicates tumor and normal cells respectively. **(c)** Isoform-specific expression of circRNAs generated from BARD1 and KRD36C. Rows show each circRNA isoform, and columns represent individual cells. **(d)** Trajectory reconstruction of epithelial cells, colored by CPM of stage-specific circRNAs. **(e)** Expression of circRNAs in the various clusters. Rows indicate primary or metastasis tumors from each patient. Source data are provided as a Source Data file.

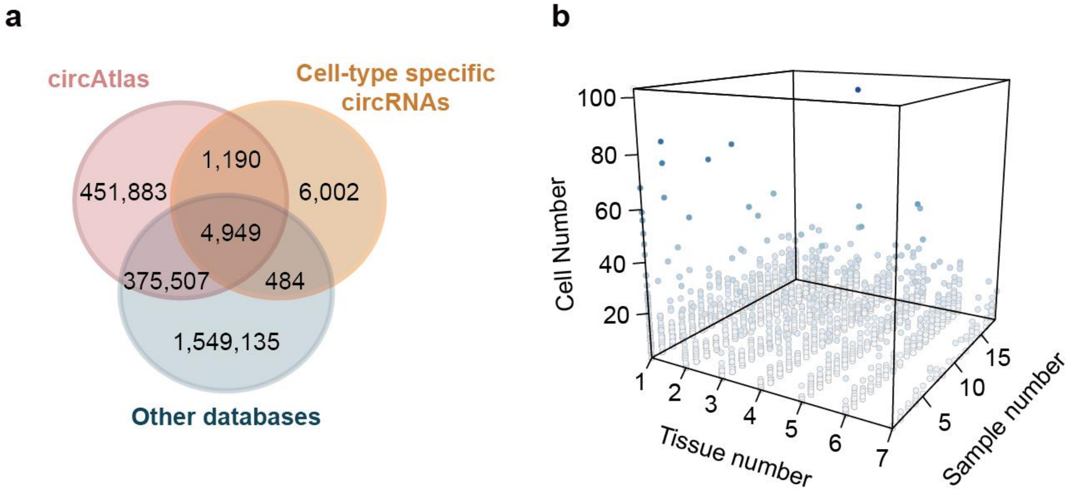

Supplementary Fig. 5 Expression of cell-type specific circRNAs in the circAtlas database. (a) The number of cell-type specific circRNAs that are reported in bulk RNA-seq based databases. **(b)** The number of expressed tissues and samples of 6,139 cell-type specific circRNAs in the circAtlas database. The z-axis represents the number of expressed cells in the scRNA-seq cohort. Source data are provided as a Source Data file.

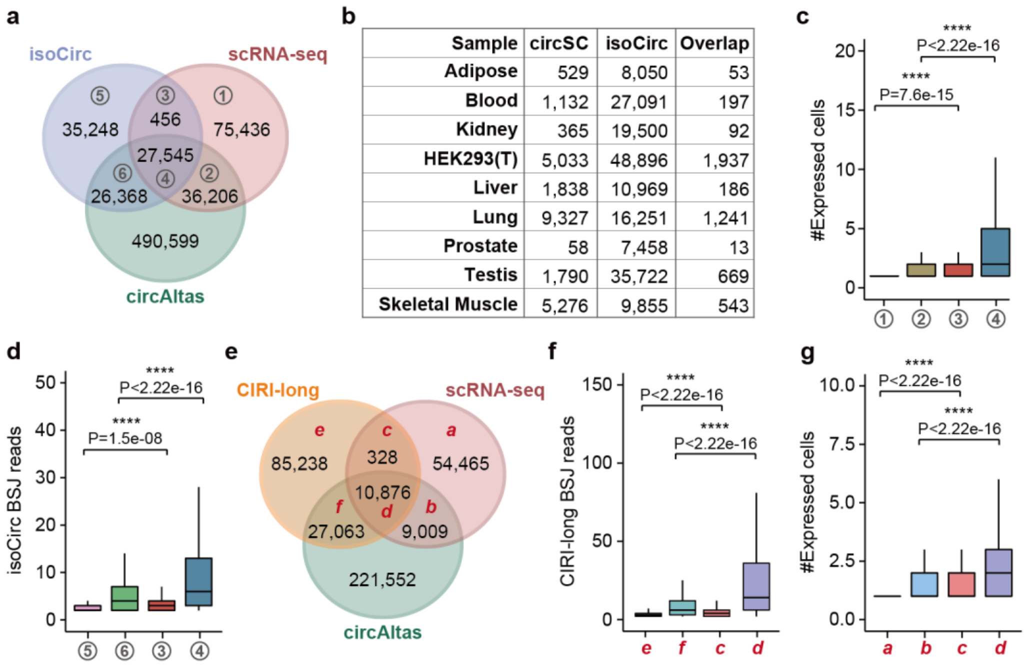

Sample	circSC	isoCirc	Overlap
Adipose	529	8,050	53
Blood	1,132	27,091	197
Kidney	365	19,500	92
HEK293(T)	5,033	48,896	1,937
Liver	1,838	10,969	186
Lung	9,327	16,251	1,241
Prostate	58	7,458	13
Testis	1,790	35,722	669
Skeletal Muscle	5,276	9,855	543

Supplementary Fig. 6 Comparison of scRNA-seq methods against isoCirc and CIRI-long. (a) Overlap of circRNAs detected in human samples from isoCirc, circAtlas and the scRNA-seq datasets. **(b)** Overlap of circRNAs detected in each tissue. **(c)** Boxplots indicate the number of expressed cells of each subset (*n* = 75,436 / 36,206 / 456 / 27,545 circRNAs). **(d)** Boxplots indicate the number of isoCirc BSJ reads of each subset (*n* = 35,248 / 26,368 / 456 / 27,545 circRNAs). **(e)** Overlap of circRNAs detected in mouse brain samples from CIRI-long, circAtlas and the scRNA-seq datasets. **(f)** Boxplots indicate the number of CIRI-long BSJ reads of each subset (*n* = 85,368 / 27,063 / 328 / 10,876 circRNAs). **(g)** Boxplots indicate the number of expressed cells of each subset (*n* = 54,465 / 9,009 / 328 / 10,876 circRNAs). All center lines in the box plots indicate the median values, and box limits indicate the upper and lower quartiles of plotted values. The upper and lower whisker indicate the largest and smallest value within the range of 1.5x IQR from the box limits. **** $P < 0.0001$, Wilcoxon rank-sum test (two-sided). Source data are provided as a Source Data file.

Supplementary Table 1. List of public bulk RNA-seq based circRNA databases.

Database name	Species	#circRNAs	Reference	URL
circAtlas v2.0	Human, Mouse	833,529	CircAtlas: an integrated resource of one million highly accurate circular RNAs from 1070 vertebrate transcriptomes	http://circatlas.biols.ac.cn
circbank	Human	140,725	Circbank: a comprehensive database for circRNA with standard nomenclature	http://www.circbank.cn
circBase	Human, Mouse	92,375	circBase: a database for circular RNAs.	http://www.circbase.org
CIRCpedia v2	Human, Mouse	177,456	CIRCpedia v2: An Updated Database for Comprehensive Circular RNA Annotation and Expression Comparison	http://www.picb.ac.cn/rnomics/circpedia/
CircRiC	Human	92,599	Comprehensive characterization of circular RNAs in ~ 1000 human cancer cell lines	https://hanlab.uth.edu/cRic/
circRNADb	Human	32,914	circRNADb: A comprehensive database for human circular RNAs with protein-coding annotations	http://reprod.njmu.edu.cn/circrnadb/circRNADb.php
CSCD	Human	1,223,114	CSCD: a database for cancer-specific circular RNAs	http://gb.whu.edu.cn/CSCD/
exoRBase	Human	57,412	exoRBase: a database of circRNA, lncRNA and mRNA in human blood exosomes	http://www.exorbase.org
MiOncoCirc v2.0	Human	227,056	The Landscape of Circular RNA in Cancer	https://mioncocirc.github.io
TSCD	Human, Mouse	284,296	Comprehensive characterization of tissue-specific circular RNAs in the human and mouse genomes	http://gb.whu.edu.cn/TSCD/
deepbase v2.0	Human, Mouse	16,758	deepBase v2.0: identification, expression, evolution and function of small RNAs, LncRNAs and circular RNAs from deep-sequencing data	http://biocenter.sysu.edu.cn/deepBase

Supplementary Table 2. RT-PCR primers and Sanger sequencing results of 12 cell-type specific circRNAs.

circRNA ID	Strand	Cell Type	#Cell	#BSJ	Forward Primer	Reverse Primer	BSJ
12:44282578|44299291	+	GABAergic Interneuron	10	282	CATGCGTTTTAT GACAGTAA	GCTCCAACTCAT TTTGACGG	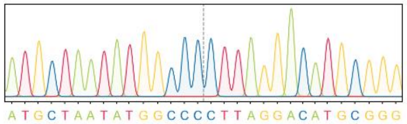
3:75358347|75397101	-	Astrocyte	9	158	GGCAAGATGGA GTTGTGG	CCTGTTTCAGGA GGGAATT	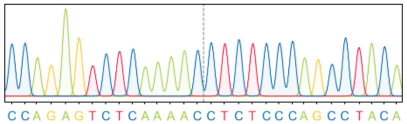
17:78237742|78355177	+	Glutamatergic excitatory	9	19	CTCTGAGTCTT GCCCTTCCG	TCGGATGGTACC ACATTCGC	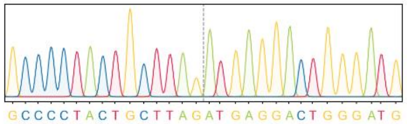
7:4028707|4029036	-	Microglia	8	136	TCCGAACGTAG TAAGACGCTG	TCCAGGCGGAC CATGTTATAC	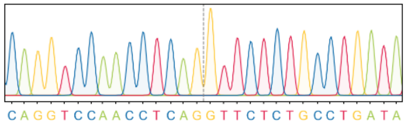
11:62351378|62398437	-	GABAergic Interneuron	8	64	AGGATGCCTAT TGAAGAAAGC	TTATGAGAAGA CAAGTAAGTGC C	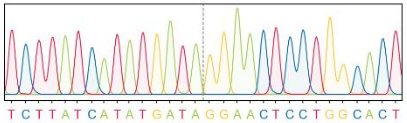
10:14004201|14066684	+	Glutamatergic Excitatory	8	21	GTTTTGTCAAA GCCAACGAT	TCACAGACTTCA CCTGGATA	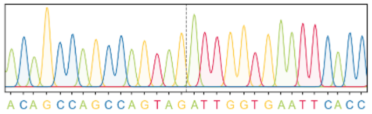
7:59420903|59529906	-	GABAergic Interneuron	8	8	TGTGACCATTC CTACTCTGA	TGGAACTGGAG AGTGATTGC	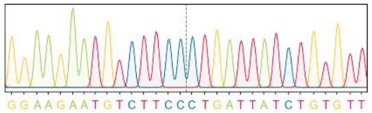
1:68560496|68740533	-	GABAergic Interneuron	6	31	AGGCTACGTCC TGGTGGCCC	GTGACTTCTCGG ATAGACCG	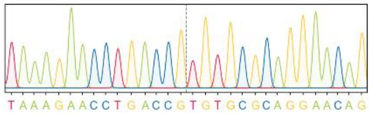

8:45759053|45763191	+	pericytes	5	18	TCCCAGCATGC CCAGTCCCT	ATATGTCTCTGC AGAATCAA	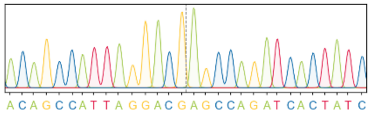
2:91548774|91550758	+	Endothelial	2	107	CGGGAGAGGTT GTATCAGGT	TCAAGGCTTCTT CATACCCACT	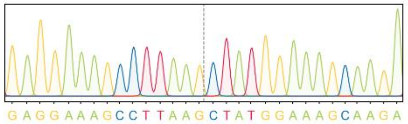
17:30135323|30153368	+	VSMC	2	67	CATGCACAGAC ACAGCTCAT	TGTCACTGGTGG TCTCTTCG	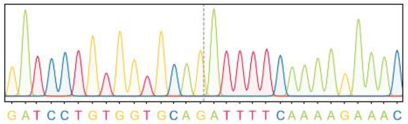
7:4028321|4029036	-	Microglia	5	76	TCCTGTAGGGA GGTGTCA	TATAAGGCTCAG TGCTCTTTT	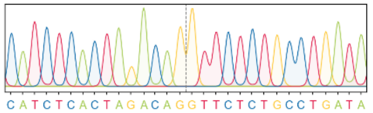

Supplementary Table 3. List of cell-type marker genes from published literature.

Tissue	Cell type	Marker genes
General	T cell	CD2, CD3D, CD3E, CD3G
	B cell	CD19,CD79A
	Macrophage	CD163,FCGR3A
	Epithelial	EPCAM,CLDN3,KRT8,CAPS, SNTN
	Fibroblast	COL1A1,COL1A2,DCN
	Endothelial	VWF,CDH5,PECAM1
	Erythrocytes	MS4A1,VPREB3
	Monocytes	CXCL10
	Adipocytes	ANPEP,ITGAX
	Alveolar cell	CLDN18, AQP4, FLOR1
	Myeloid cell	CD14, LYZ
	Neutrophils	CSF3R, S100A8, S100A9
	Follicular dendritic cell	FDCSP
	Granulocytes	CEBPE,HDC,MS4A2
	Keratinocyte	KRT1,KRT14
	Smooth muscle	ACTA2,ACTG2,MYH11
	Neuroendocrine	CHGA,SCG5,CHGB
	Cholangiocyte	MUC5B
	Plasma	JCHAIN,IGKV1-12
	Satellite	DLK1
	Red blood cell	CD235a
	Mast cell	GATA2, TPSAB1, TPSB2
	FibSmo	KRT17
	Myeloid	MKI67,CALB2
	Dendritic	IL3RA,GZMB
Brain	Astrocyte	GFAP,SLC1A2,AQP4,GJA1,GJB6,SLC4A4,SLC39A12,FABP7
	Endothelial	CD34,VWF,FTL1,RGS5,PTPRB,PALMD,APOLD1,NOSTRIN,ESAM,CLDN5,PECAM1
	Microglia	ITGAM,CX3CR1,CCL3,CSF1R,P2RY12,CCL4,IBA1,C1QA,LAPTM5,C1QB,C1QC,AIF1,HLA-DRA,TYROBP,CTSS,TREM2,TMEM119,CD11B
	Oligodendrocyte	MOG,MBP,MAG,OPALIN,MOBP,CNP,CLDN11,PLP1,O4
	OPC	OLIG1,OLIG2,PDGFRA,VCAN,SOX10,GPR17
	GABAergic interneuron	GAD1,GABBR1,GABBR2,GAD2,PBX3,DLX1,DLX2,'DLX5'
	Glutamatergic excitatory	RORB,FEZF2,THEMIS,LAMP5,LTK,GLS,GRIN1,GRIN2B,SLC17A7,SLC17A6
	VLMC	OGN,LUM,DCN
	Tancyte	RAX,CX43

	Macrophages	APOE,MS4A7,MS4A6C,LYZ2,TGFBI,CCL12
	Ependymocytes	CCDC153,AK7,PITO
	Intermediate progenitors	EOMES
	Medium spiny neuron	PITX3,SLC6A3
	Mural	PDGFRB,RGS5
Colon	Enterocytes	KRT20,CDH17,SLC26A3
	Goblet cells	TFF3,CLCA1,SPINK4
	Paneth cells	SPIB,CA7,BSET4
Kidney	Collecting duct cell	AQP2,PVALB,TMEM213
	Collecting duct principal cell	AQP2
	Proximal tubular cell	SLC22A8,TMEM174,MIOX,NAT8
	Erythroid cell	CA1,ALAS2
	Mesenchymal cell	DCN,RELN,FSCN1
Liver	Cholangiocytes	KRT19,MUC5B,ELF3
	Erythroid cell	CA1,ALAS2
	Hepatocytes	ALB,FGG,FGA,HPX
	Kupffer cells	MARCO,S100A8,S100A9
Lung	Bronchial epithelium, basal cells	KRT17,KRT5,S100A2
	Alveolar cells	CAV1,EMP2,AGER,SFTPA2,NAPSA,LAMP3
Pancreas	Pancreatic endocrine cells	INS,GCG,PPY
	Ductal cells	CRP,CFTR,AQP1
	Exocrine glandular cell	AMY2A,CPA1,CELA3A
Small intestine	Enterocytes	SI,MUC17,ALPI
	Goblet cells	TFF3,CLCA1,SPINK4
Testis	Spermatocytes	SPO11,SYCP3,TOP2A
	Late spermatids	TNP1,PRM2,SPATA3
	Spermatogonia	SOX4,UTF1,KIT
	Peritubular cell	ACTA2,ACTG2,MYH11
Retina	Retinal ganglion cell	NEFL, TPM1, MYL9
	Horizontal cell	ONECUT1, ONECUT2

Supplementary Table 4. List of abbreviations for cell types mentioned in this study.

Abbreviation	Name	Abbreviation	Name
ADPC	Adipocytes	SPCL	Late spermatids
ALVC	Alveolar cell	MAC	Macrophages
ASC	Astrocyte	MSN	Medium spiny neuron
BC	B cell	MESA	Mesangial cell
BEPI	Bronchial epithelium	MESC	Mesenchymal
BRUC	Brush cell	MG	Microglia
CHOL	Cholangiocyte	MYOC	Myoblasts
CDRC	Chondrocyte	NPC	Neural progenitor cell
CDC	Collecting duct cells	OLG	Oligodendrocyte
CDPC	Collecting duct principal cell	OPC	Oligodendrocyte progenitor cell
DC	Dendritic	PERC	Pancreatic endocrine cell
DUCT	Ductal cell	PC	Pericytes
EC	Endothelial	PERI	Peritubular cell
ENTC	Enterocytes	PLAC	Plasma cell
EEC	Enteroendocrine cell	PRTC	Proximal tubular cells
EPIC	Epithelial	RBC	Red blood cell
ERYC	Erythroid cell	RGC	Retinal ganglion cells
EXGC	Exocrine glandular cell	SECR	Secretory cell
FEC	Fenestrated cell	SKMC	Skeletal muscle satellite cell
FB	Fibroblast	SMC	Smooth Muscle
GABA	GABAergic interneuron	SPC	Spermatocytes
GLUT	Glutamatergic excitatory	SPG	Spermatogonia
GC	Goblet cells	TC	T cell
HEAC	Henle ascending limb epithelial cell	VLMC	Vascular leptomeningeal cell
HEPA	Hepatocytes	VSMC	Vascular smooth muscle cell
HZC	Horizontal cells	KERC	Keratinocytes
IP	Intermediate progenitors	BPLC	Bipolar
ICSC	Intestinal crypt stem cell	MELC	Melanocytes
KUPC	Kupffer cells	GRAN	Granulocytes
