# Supplementary material for: Exploring the cellular landscape of circular RNAs using full-length single-cell RNA sequencing
Source: Nat Commun. 2022 Jun 10;13:3242. doi: 10.1038/s41467-022-30963-8 (PMC9187688; doi:10.1038/s41467-022-30963-8)
Supplement: Supplementary file 4 — Reporting Summary [file 41467_2022_30963_MOESM4_ESM.pdf]

nature portfolio

Corresponding author(s): Fangqing Zhao

Last updated by author(s): May 8, 2022

Reporting Summary

Nature Portfolio wishes to improve the reproducibility of the work that we publish. This form provides structure for consistency and transparency in reporting. For further information on Nature Portfolio policies, see our Editorial Policies and the Editorial Policy Checklist.

Statistics

For all statistical analyses, confirm that the following items are present in the figure legend, table legend, main text, or Methods section.

n/a	Confirmed	
☐	☒	The exact sample size (*n*) for each experimental group/condition, given as a discrete number and unit of measurement
☐	☒	A statement on whether measurements were taken from distinct samples or whether the same sample was measured repeatedly
☐	☒	The statistical test(s) used AND whether they are one- or two-sided<br>*Only common tests should be described solely by name; describe more complex techniques in the Methods section.*
☒	☐	A description of all covariates tested
☐	☒	A description of any assumptions or corrections, such as tests of normality and adjustment for multiple comparisons
☐	☒	A full description of the statistical parameters including central tendency (e.g. means) or other basic estimates (e.g. regression coefficient) AND variation (e.g. standard deviation) or associated estimates of uncertainty (e.g. confidence intervals)
☐	☒	For null hypothesis testing, the test statistic (e.g. *F*, *t*, *r*) with confidence intervals, effect sizes, degrees of freedom and *P* value noted<br>*Give P values as exact values whenever suitable.*
☒	☐	For Bayesian analysis, information on the choice of priors and Markov chain Monte Carlo settings
☒	☐	For hierarchical and complex designs, identification of the appropriate level for tests and full reporting of outcomes
☐	☒	Estimates of effect sizes (e.g. Cohen's *d*, Pearson's *r*), indicating how they were calculated

Our web collection on statistics for biologists contains articles on many of the points above.

Software and code

Policy information about availability of computer code

Data collection	Public RNA-seq datasets were downloaded using sratoolkit 2.9.4
Data analysis	Full-length scRNA-seq data were analyzed using HISAT2 (v2.0.5), StringTie (v1.2.4). Quality control were performed using Scater (v1.18.6). Seurat (v.4.0.2) were used for downstream analysis. Cell types were annotated using SingleR (v1.4.1) and CopyKAT (v1.0.4) workflow, and pseudo-time trajectory analysis were performed using Monocle2 (v2.8.2). The circRNA expression at single-cell level were estimated using the bwa (v0.7.12), CIRI2 (v2.0.6) and CIRIquant (v1.1) pipeline. The time-series cluster were performed using Mfuzz (v2.50.0). Gene ontology analysis were performed using ClusterProfiler (v4.0) and Enrichr. The bulk RNA-seq deconvolution were performed using the R version of CIBERSORT.

For manuscripts utilizing custom algorithms or software that are central to the research but not yet described in published literature, software must be made available to editors and reviewers. We strongly encourage code deposition in a community repository (e.g. GitHub). See the Nature Portfolio guidelines for submitting code & software for further information.

Data

Policy information about availability of data

All manuscripts must include a data availability statement. This statement should provide the following information, where applicable:

- Accession codes, unique identifiers, or web links for publicly available datasets
- A description of any restrictions on data availability
- For clinical datasets or third party data, please ensure that the statement adheres to our policy

The cellular expression results of circRNAs identified in this study are available at the “circSC” module in circAtlas (http://circatlas.biols.ac.cn). The RNA-seq datasets used for circRNA identification are listed in the Supplementary Data 1. Source data have been deposited in the Zenodo repository (https://zenodo.org/

record/6528434).

Field-specific reporting

Please select the one below that is the best fit for your research. If you are not sure, read the appropriate sections before making your selection.

- [x] Life sciences
- [ ] Behavioural & social sciences
- [ ] Ecological, evolutionary & environmental sciences

For a reference copy of the document with all sections, see nature.com/documents/nr-reporting-summary-flat.pdf

Life sciences study design

All studies must disclose on these points even when the disclosure is negative.

Sample size	The full-length scRNA-seq data from 171 studies involving 58 different human and mouse tissues or cell types were collected. The sample size is determined by the number of public available datasets. No statistical method was used to predetermine sample size. No data were excluded from the analyses and the all analysis were not randomized. A total of 40604 human and 131533 mouse single cells were kept after quality control, providing enough statistical power for inferring the circRNA expression landscape at single-cell resolution.
Data exclusions	No data exclusions was involved in this study.
Replication	Sanger sequencing was performed to validate the PCR products in this study. No experimental validation was involved in this study.
Randomization	Samples were grouped based on their tissue types, and no randomization was involved in this study.
Blinding	We only included public scRNA-seq data and performed data analysis, no clinical trials or other experimental analysis were performed in this study. Thus no blinding group allocation was involved in this study

Reporting for specific materials, systems and methods

We require information from authors about some types of materials, experimental systems and methods used in many studies. Here, indicate whether each material, system or method listed is relevant to your study. If you are not sure if a list item applies to your research, read the appropriate section before selecting a response.

Materials & experimental systems

n/a	Involved in the study	
x		Antibodies
x		Eukaryotic cell lines
x		Palaeontology and archaeology
x		Animals and other organisms
x		Human research participants
x		Clinical data
x		Dual use research of concern

Methods

n/a	Involved in the study	
x		ChIP-seq
x		Flow cytometry
x		MRI-based neuroimaging
